# Conservation Planning for Coral Reefs Accounting for Climate Warming Disturbances

**DOI:** 10.1371/journal.pone.0140828

**Published:** 2015-11-04

**Authors:** Rafael A. Magris, Scott F. Heron, Robert L. Pressey

**Affiliations:** 1 Australian Research Council Centre of Excellence for Coral Reef Studies, James Cook University, Townsville, Queensland, Australia; 2 National Oceanic & Atmospheric Administration Coral Reef Watch, Townsville, Queensland, Australia; 3 Physics Department, Marine Geophysical Laboratory, College of Science, Technology and Engineering, James Cook University, Townsville, Queensland, Australia; The University of Hong Kong, HONG KONG

## Abstract

Incorporating warming disturbances into the design of marine protected areas (MPAs) is fundamental to developing appropriate conservation actions that confer coral reef resilience. We propose an MPA design approach that includes spatially- and temporally-varying sea-surface temperature (SST) data, integrating both observed (1985–2009) and projected (2010–2099) time-series. We derived indices of acute (time under reduced ecosystem function following short-term events) and chronic thermal stress (rate of warming) and combined them to delineate thermal-stress regimes. Coral reefs located on the Brazilian coast were used as a case study because they are considered a conservation priority in the southwestern Atlantic Ocean. We show that all coral reef areas in Brazil have experienced and are projected to continue to experience chronic warming, while acute events are expected to increase in frequency and intensity. We formulated quantitative conservation objectives for regimes of thermal stress. Based on these objectives, we then evaluated if/how they are achieved in existing Brazilian MPAs and identified priority areas where additional protection would reinforce resilience. Our results show that, although the current system of MPAs incorporates locations within some of our thermal-stress regimes, historical and future thermal refugia along the central coast are completely unprotected. Our approach is applicable to other marine ecosystems and adds to previous marine planning for climate change in two ways: (i) by demonstrating how to spatially configure MPAs that meet conservation objectives for warming disturbance using spatially- and temporally-explicit data; and (ii) by strategically allocating different forms of spatial management (MPA types) intended to mitigate warming impacts and also enhance future resistance to climate warming.

## Introduction

Rapidly increasing concentrations of anthropogenic greenhouse gases that induce climate change are triggering dramatic declines in coral reefs worldwide [1]. Several factors are thought to be responsible for these declines, including elevated sea-surface temperature, sea-level rise, effects on reef calcification, and solar radiation [2]. Increases in sea temperature have led to shifts in species’ phenologies [3], rates of reproductive success [4], metabolic rates [5], and geographic ranges [6]. There have also been substantial shifts in the abundance and composition of coral communities affected by bleaching events [7]. In combination with more localized stresses, such as overfishing and degraded water quality, unprecedented thermal stress impacts could undermine significant investments in protection of coral reefs over recent decades [8]. The rapid pace of climate warming is likely to increase damage to coral reefs; consequently, improved understanding of proactive conservation strategies is pivotal to sustainably managing marine populations.

Reef-building corals are particularly vulnerable to rising sea temperatures and are among the most sensitive organisms to climate change [1]. Corals under temperature stress lose the ability to synthesize protective sunscreens, making them more sensitive to sunlight [9]. In addition, reef-building corals have relatively long generation times and low genetic diversity, a combination that slows adaptation to environmental changes [1]. Although adaptive responses to thermal stress could increase with climate warming [10], adaptive capacity might include a shift to symbiont species with a higher thermal tolerances, which can still be considered a kind of reef degradation [11]. Corals already live near their thermal limits [7]. Temperatures that exceed normal summer maxima by only 1°C are enough to cause coral bleaching, and prolonged high temperatures over large areas can lead to extensive mortality [12]. Disruption of coral growth and composition can also be protracted because rates of recovery vary considerably across species and environmental conditions; such disruption is linked to the recurrence of mortality events, and other concurrent stressors [13–15].

Understanding where and how to mitigate warming impacts, and thereby manage the resilience of coral reef ecosystems, is a central concern of conservation planning [16]. However, conservation plans for coral reefs that account for warming disturbances often neglect the spatial and temporal variability of thermal impacts [8, 17]. For example, design of marine protected areas (MPAs) within the context of climate change frequently uses simple ‘rules of thumb’, such as selecting multiple, spatially separate samples of the same reef type (replication) to be protected as a risk-spreading approach. [18]. As a consequence, observations of MPAs mitigating temperature-driven coral loss are limited [19]. Despite this lack of empirical evidence, MPAs might help to alleviate associated impacts by removing or reducing non-climate stressors [20], protecting sites that can promote re-colonization of extirpated populations elsewhere [21], or accelerating recovery from uncontrollable disturbances [22].

Previous studies have suggested a variety of quantitative methods that incorporate thermal-related impacts into marine planning to design effective strategies for conservation in a changing climate. Most of the prioritization approaches are based on the use of historical satellite data on climate variability [21, 23–25], predicted climatic regime [8], or a combination of both [26, 27]. However, these studies have not fully integrated historical and predictive climate variability within MPA design tools (but see Levy and Ban, [26]) to identify high-priority areas where coral reefs can be protected both now and in the future. Here, we propose an approach to MPA design that includes explicit spatial and temporal information on warming impacts to determine spatial configurations of MPAs that meet conservation objectives related to climate change. Our approach is applicable when MPAs are designed to simultaneously achieve long-term objectives considering two time frames (historical and future) as opposed to developing MPAs that need to be moved as disturbance regimes shift.

To account for the challenges around MPA design in the context of global warming, the approach described here also offers the opportunity to include a more comprehensive set of management actions than simple generic protection. Rather than focusing only on the dominant approach of protecting areas relatively unaffected by global warming (i.e., thermal refugia, see Ban et al. [28] and Levy and Ban [26]), we show that MPAs can be strategically located for diverse management actions that also cover sites most suitable for mitigation of cumulative stresses, facilitation of adaptive processes, and future resistance to warming.

This study evaluates the suitability of MPA networks to protect coral reefs under historical and future climate conditions. First, we identify the relative exposures of reefs to different historic and future thermal-stress regimes using measures of chronic and acute stress. Chronic stress corresponds to the long-term rate of warming and can be considered in relation to the ability of organisms to acclimatize [29]. Acute stress, occurring over shorter time-scales, can result in sporadic bleaching events that impair ecosystem function [7]. Second, we formulate indicative conservation objectives for thermal-stress regimes that can be set in the decision-making process to boost resilience and aid the development of a climate-resilient system of MPAs. Finally, we assess the gaps in the representation of thermal-stress regimes by an existing system of MPAs and identity priority areas where additional protection would capture complementary thermal-stress regimes, thereby reducing the risk of establishing climatically unrepresentative reserves.

## Materials and Methods

**Fig 1:**
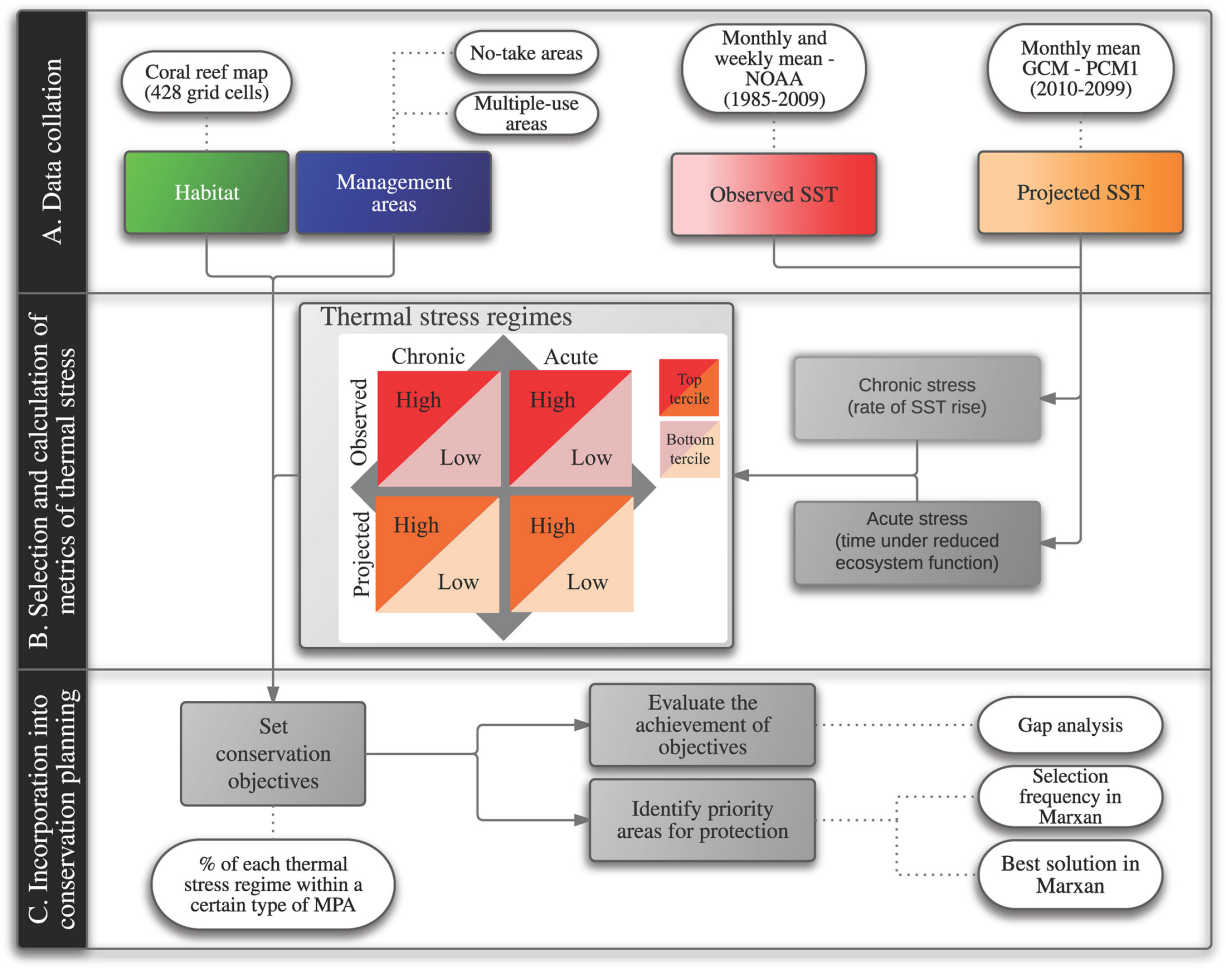
Methods of this paper divided into three major phases. (A) Data collation involved acquisition of habitat data (green box), boundaries of marine protected areas (MPAs) (blue box), and observed and projected data (red and orange boxes, respectively) on sea-surface temperature (SST). In the selection and calculation of metrics of thermal stress (B), we derived metrics of chronic and acute stress from observed and projected datasets and combined them to define thermal-stress regimes. Regimes were delineated based on upper and lower terciles labelled as “high” (highest 33% of values, dark red or orange) and “low” (lowest 33% of values, light red or orange), respectively. The incorporation of warming disturbances into conservation planning (C) consisted of setting conservation objectives for each thermal-stress regime, evaluating their achievement in existing MPAs, and identifying priority areas that would achieve unmet objectives. Arrows in gray indicate the flow of information and lighter boxes linked by dashed lines depict types of data or analyses involved in each step.

Our study comprised a three-step procedure for incorporating potential impacts of warming disturbances into MPA design, using detailed information on historical and future thermal stress (Fig 1). The steps were: (A) data collation, (B) selection and calculation of metrics of thermal stress, and (C) incorporation of warming disturbances into marine conservation planning. The method considered both the magnitude and duration of climate-related exposure to stress and the ability of coral reefs to withstand such exposure. We included historical data (henceforth referred to as ‘observed’ data) and future projections of climatic conditions (henceforth referred to as ‘projected’ data) because we aimed to analyze not only those areas that have already experienced changes but also those most likely to be affected by future climate-related disturbances.

Our study area covers Brazilian coral reefs (within ~4°30'N– 51°37'W to ~18°30'S– 24°38'W), which are a priority for marine conservation in the southwestern Atlantic Ocean. In Brazil, warming temperatures appear to be driving both coral bleaching [30] and the incidence of coral diseases [31]. Bleaching events have been recorded predominantly on the eastern reefs (Bahia state) since 1993 [32]; there is a paucity of field data on bleaching in other areas (but see [33, 34]). In the years in which major events occurred (1997/1998, 2002/2003, 2009/2010), bleaching was fairly widespread, spanning about 500 km of coast, and causing significant coral mortality and/or sublethal effects [30, 35, 36]. Although Brazilian reefs are thought to conform only partially with global patterns of bleaching [32] sea temperature has been identified as a key driver of bleaching events [37]. Despite high relative representation of Brazilian coral reefs within MPAs [38], reef degradation has not been mitigated or prevented by local management [34, 39].

### Data collation

**Fig 2:**
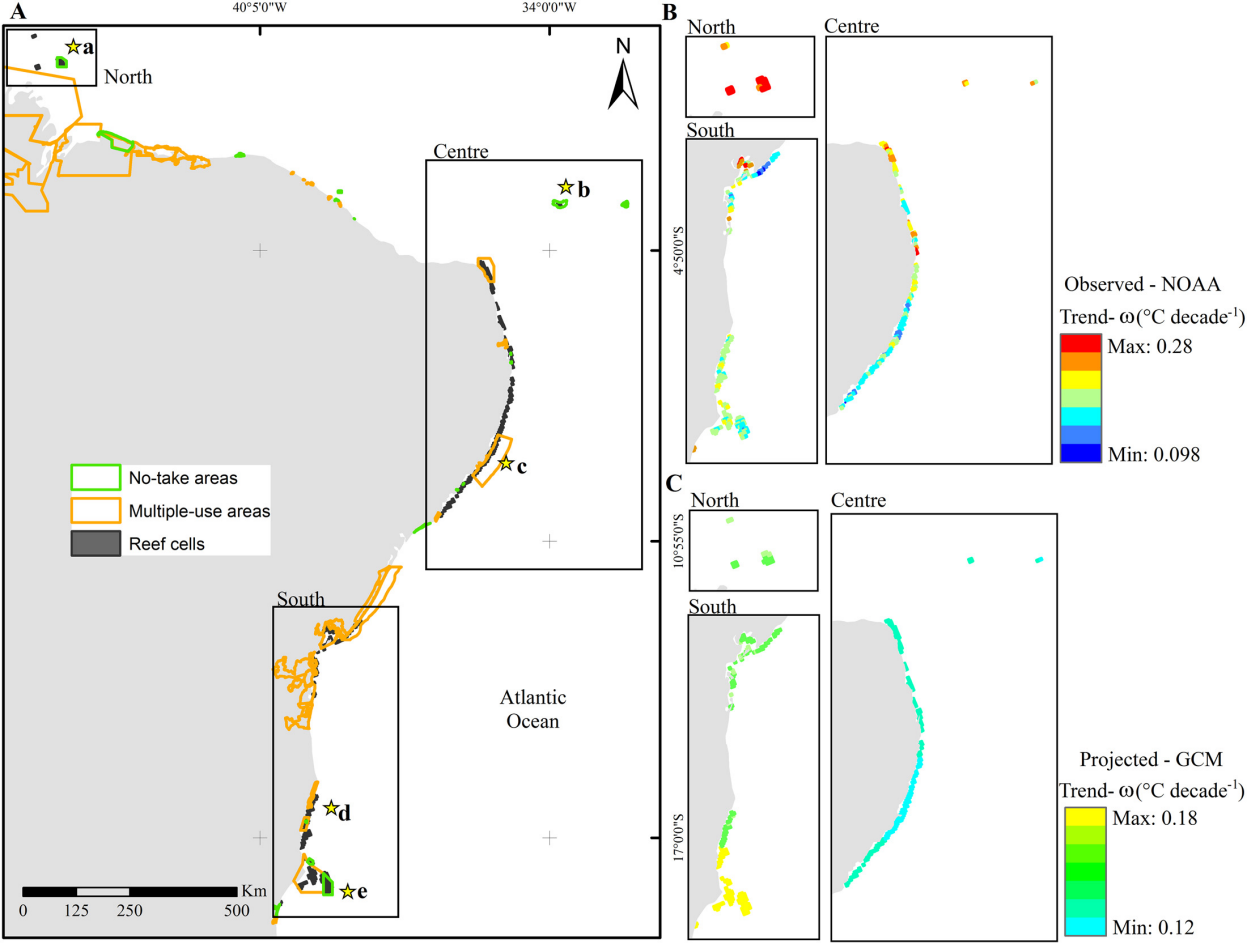
The study area and the chronic stress metric. (A) Sectors (northern, central, southern), reef cells (n = 428), and the existing MPA boundaries along the Brazilian coast. MPAs are classified according to their main management categories: no-take areas and multiple-use areas. Letters a-e with stars denote approximate locations of reef cells selected to depict temperature variability (see Fig 6). (B) Decadal SST trends describe observed chronic stress for each reef cell from NOAA satellite data. (C) Decadal SST trends describe projected chronic stress for each reef cell, downscaled from PCM1 general circulation model output.

The total area of coral reefs in Brazil is ~900 km^2^ occurring in three distinct geographical sectors: northern, central, and southern (Fig 2A). To align with the temperature data (see below), we identified ~4 x 4 km grid cells that contain coral reefs (habitat data). The resulting 428 reef cells were used to summarize results for thermal stress and selection of potential new MPAs. We also compiled a dataset on existing MPAs along the Brazilian coast (Fig 2A) with their legal boundaries [38]. Here, we refer to a system of MPAs as an array of individual MPAs encompassing a wide range of management types and levels of protection. For the purposes of this study, we consider two management types: (i) no-take areas, where ecosystems should be preserved in a state undisturbed by extractive activities; and (ii) multiple-use areas, with objectives to promote the sustainable use of the marine environment by a wide variety of users, with extractive activities permitted but regulated.

For historical analysis, we acquired data on sea-surface temperature (SST) from the National Oceanic and Atmospheric Administration (NOAA) Pathfinder Project (http://pathfinder.nodc.noaa.gov) [40]. Version 5.0 data at ~4 km spatial resolution spanning the period 1985–2009 were retrieved for our study area. The dataset comprised a lengthy, accurate, and consistent set of records with high spatial resolution [40]. The information in these records has contributed to a wide range of marine applications related to conservation [41–43]. We used only night-time values because they are most relevant for coral habitats [44]. Weekly composites of only high-quality values were produced and data gaps filled, following the method of Heron et al. [45].

For our analysis of future projections, we used global monthly SST output (2010–2099) by the Parallel Climate Model PCM1, which is a General Circulation Model (GCM) developed by the National Center for Atmospheric Research (NCAR) for the Intergovernmental Panel on Climate Change, Fourth Assessment (IPCC AR4). PCM1 outputs were acquired from the World Climate Research Program Coupled Model Intercomparison Project Phase 3 (WCRP CMIP) multi-model database. The model has an oceanic resolution of ⅔° × ½° and has the lowest climate sensitivity (1.7°C) among the 23 different IPCC models that predict impacts of climate change on ocean temperature [46]. This model was selected because it represents a lower bound for projected ocean warming and has performed relatively well in a global prediction of bleaching frequency [47]. The output files were selected for the A1B emission scenario, which represents business-as-usual greenhouse gas emissions over the current century; under this scenario, atmospheric concentration of greenhouse gases will reach 720 ppm by 2100 and stabilize at this level. The scenario describes increases in concentration of greenhouse gases attributable to expected human population growth and industrial development. A1B scenarios are mid-line within the A1 scenario family for carbon dioxide output and economic growth [48].

A key limitation revealed by spectral analysis is that many GCMs under- or over-represent variability about their means (the baselines from which predictions are produced) or seasonal cycles, reducing their capacity for projections of coral bleaching [49] and for undertaking more informed conservation planning [50]. Importantly, van Hooidonk and Huber [51] detected PCM1 over-prediction of some components of climate, such as the variability of the tropical ocean seasonal cycle and ENSO in a comparison with observations of SST data averaged over all global reef locations. Recognizing this limitation in attempts to realistically represent SST variability and accurately predict bleaching for some of our reef locations, we applied a bias-removal technique following the method described by Dunne et al. [52] to make the forecasts more consistent in intensity and timing (S1 File). This involved statistical downscaling of the coarse spatial resolution of the GCM projections to the fine resolution of historical satellite data (~4 km) and included setting the mean and variance of the projections to those of the observational data (using retrospective projections covering 1985–1999, i.e. the training period; see Figure A in S1 File) [52]. Although the downscaling process performed here does not resolve local-scale features such as eddies, high-resolution (4 km) observations and projections of SST are suitable for MPA design and management [50].

While other observed and projected datasets have since become available, the SST datasets described above were the most-recently available at the time of analysis.

### Selection and calculation of thermal stress metrics

Myriad measures of thermal stress could be used in marine conservation planning as predictors of coral-reef resilience in the face of climate-related disturbances [8, 19, 26–28, 43, 53–57]. Two indicators—SST trend and Degree Heating Weeks (DHWs)—emerged from previous studies as realistic and reliable ways of detecting detailed spatial and temporal patterns in impacts of temperature on coral-reef ecosystems [56, 58]. SST trends and DHWs were used to determine the spatial distribution of chronic and acute thermal stress, respectively, across our study area. These metrics allowed us to accurately compare different thermal-stress regimes based on both observed and projected SST datasets (Fig 1B).

#### Chronic thermal stress

Chronic thermal stress was measured as the estimated rate of SST warming following Chollett et al. [58] and Weatherhead et al. [59]. Observed data were composited to monthly resolution (from weekly) for calculation of trends. We used non-linear mixed effect models (package nlme in R) because they are among the most robust statistical models for the detection of reliable trends in SSTs [58] and are widely used to detect trends in environmental data [60, 61]. The basic structure of the model is:

$$SST_{t}=\;\mu +\;S_{t}+\omega t/12+N_{t}$$ (1)

where, *SST*_*t*_ at a given time *t* (in months) is a function of a constant term *μ*, a seasonal component *S*_*t*_, a linear trend *ω* of the rate °C *yr* ^−1^ and residuals *N*_*t*_, which is an assumed autoregressive of order one (AR-1 autocorrelation form). This structure allowed us to account for some variability in the time series, such as seasonality and serial correlation, which influence the magnitude and significance of the calculated trends [59]. By using monthly means derived from satellite observations and GCM outputs, we quantified the overall trend in SST (in °C *decade* ^−1^) to estimate long-term, chronic thermal stress in both observed and projected data for each reef cell.

#### Acute thermal stress

Acute thermal stress was based on DHWs, a well-established indicator of coral bleaching that combines both intensity and duration of warm anomalies in relation to ecological thresholds [62]. Empirical evidence indicates a strong relationship between bleaching / mortality and level of heat stress: some coral bleaching is predicted to occur when the DHW value exceeds 4°C-weeks; widespread mortality is expected when it reaches 8°C-weeks [62]. We assessed both spatial distribution of annual maximum DHW and number of bleaching-level stress events (DHW ≥ 4°C-weeks) per decade. Reef recovery, and the sustained provision of various ecosystems goods, might not occur when two or more bleaching-level events occur per decade [63, 64].

DHWs were derived from two sources: (1) observed: weekly composites of satellite SST data, and (2) projected: monthly mean SST from PCM1 outputs. We calculated observed DHWs by taking the sum of the positive SST anomalies that exceeded the maximum climatological temperature (warmest long-term monthly average) by at least 1°C through a 12-week window [27, 56]. We subsequently recorded annual maximum DHWs for each reef cell to provide the basic historical metrics of acute stress.

To predict the occurrence of acute coral bleaching events in the period 2010–2099, we first calculated Degree Heating Months [47] by summing positive modeled SST anomalies compared with the maximum monthly SST provided by satellite climatology (cf. Donner et al. [54]) through a three-month rolling window. Annual maximum DHM values were recorded for all grid cells and converted into DHWs (using the relationship in Donner et al. [65]).

**Fig 3:**
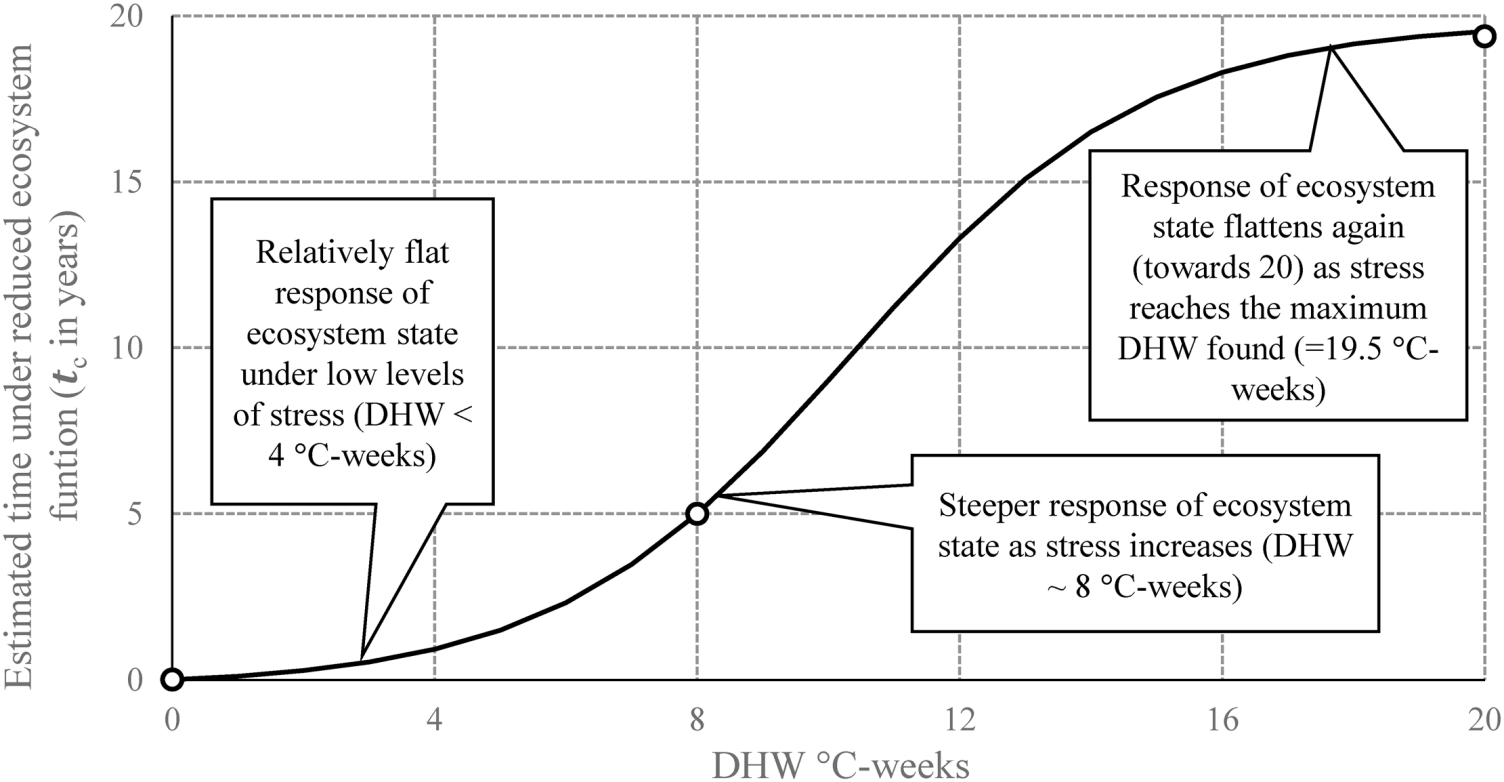
Conceptual illustration of the effects of acute thermal stress on coral-reef ecosystem state described by a logistic function. Empty circles indicate values used to fit the model. The form of this function assumes that the time that coral reefs spend with reduced ecosystem function (capacity to grow, repair, and reproduce), *t*_*c*_, is short at low DHW values because we expect that corals would recover quickly (within one year). This is followed by a steeper increase in *t*_*c*_; when widespread mortality begins (DHW reaches 8°C-weeks), the time that corals would spend recovering increases rapidly as bleaching-level events intensify above this level. When almost the entire community is extirpated over large spatial scales (above about 16°C-weeks), we expect to have small increments of *t*_*c*_ with increasing DHW for the ecosystem as a whole because only stress-tolerant species that can withstand greater acute disturbance are present. The function then reaches an upper bound (i.e., in the formulae—asymptotic value—equal to 20 years) which is the maximum time required to regenerate a fully functional ecosystem after bleaching causes massive mortality and extirpates all organisms.

We sought to characterize a single value for acute disturbance at each reef cell that took into account both frequency and intensity of bleaching-level stress events through each record. Summing individual values for thermal exposure through the time-series would not distinguish between the dramatic difference to ecosystem impact from infrequent severe events (e.g., three events with DHW of 6°C-weeks, totaling 18°C-weeks) as compared with frequent moderate events (e.g., nine events with DHW of 2°C-weeks, also totaling 18°C-weeks). We developed a logistic function model to estimate the amount of time a given reef cell is under reduced ecosystem function (capacity to grow, repair, and reproduce) after each discrete disturbance event based on DHW values (*t*_*c*_ in Fig 3). Values of *t*_*c*_ are short after exposure to low DHW values: natural communities are highly resilient to disturbance under low levels of stress and corals would move back to a natural steady state quickly [22]. The response of *t*_*c*_ then increases rapidly from the onset of bleaching-level stress (DHW = 4°C-weeks [62]). At this level, selective mortality following disturbance has a direct impact on the structure and composition of the coral community, by changing the absolute and relative abundances of coral species and filtering out less tolerant species [66]. As further thermal disturbance degrades the ecosystem and colonies are decimated over large spatial scales, the function flattens because only species with stress-tolerant life histories are present. The function then reaches an upper bound where no additional time is needed as acute thermal stress has extirpated all organisms. This model represents the conventional view of resilience (see Bellwood et al. [22]) and provides a realistic relationship between acute stress events and recovery of ecosystem function *ceteris paribus*.

Scientific evidence indicates that coral reefs that have experienced severe acute events with high associated coral mortality (DHW = 8°C-weeks) require at least 5 years to shift back to their original condition; 20 years is defined as the longest period required for returning to an unaltered state once coral mortality has resulted in complete degradation of the reef ecosystem [13, 63, 64, 67] and coincides with our maximum DHW found. We used this information to empirically fit the logistic function:

$$t_{c}=\frac{c}{1+ae^{-bx}}+d$$ (2)

where *t*_*c*_ is the estimated time under reduced ecosystem function following exposure to annual maximum DHW of *x* for each year; *a*, *b* and *c* are parameters; and *d* = −*c*/1+*a*. The values for controlling parameters *a* and *b* were determined by an experimental curve-fitting procedure and *c* was the asymptotic value or the maximum observed time to fully shift back to unaltered state after bleaching caused massive mortality.

Our acute stress metric accumulated the function values above to calculate the total amount of time that a given reef cell would spend recovering from acute events with reduced ecosystem function across each of the observed and projected time series as an estimation of total impact from past and future short-term events, respectively. This employed an assumption that each subsequent acute event contributed additively to reef degradation, regardless of how close to full recovery a given grid cell might be since the prior acute disturbance. Importantly, the underlying concept of our metric does not take into account any adaptation or acclimation by corals and their symbionts to increasing thermal stress. Accordingly, our metric is a pessimistic estimate of the amount of time in which each reef cell was prevented from returning to its unaltered state. Values are presented as number of years per decade, allowing direct comparison between observed and projected indices.

### Incorporating warming disturbances into marine conservation planning

#### Thermal-stress regimes

**Fig 4:**
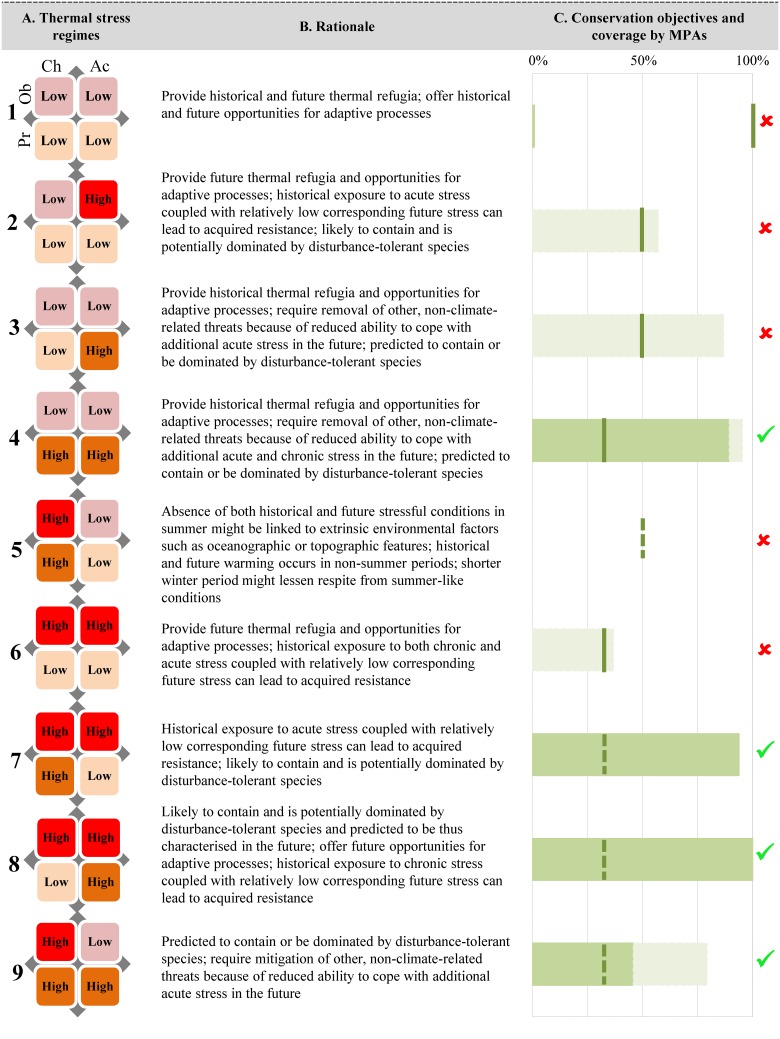
Thermal-stress regimes. The nine thermal-stress regimes defined within our study area (A), defined by combinations of high and low values for observed (Ob) and projected (Pr) chronic (Ch) and acute (Ac) stress. The rationale for management of each regime is summarised in (B). (C) Conservation objectives (dark green vertical lines) for each thermal-stress regime and their coverage by MPAs (green bars). Objectives prescribe the percentage of the total extent of reef cells in the regime (100%, 50%, or 30%) requiring management and the type of management required (no-take—solid green lines; multiple-use areas—dashed green lines). Horizontal bars indicate the percentage of each thermal-stress regime covered by the two types of MPAs: no-take MPAs are indicated by green bars; multiple-use MPAs are indicated by light green bars. The green checkmark symbol indicates that the conservation objective has been fully achieved in both extent and management type; the red “x” indicates that the conservation objective has not been attained. Objectives were formulated for explictiness in the design of MPAs to account for resilience to warming impacts, considering supporting evidence in the literature (see S2 Fig for further details about rationales to protect all regimes).

We began our exercise by partitioning the planning region into distinct disturbance regimes. For this step, metrics of both chronic and acute thermal stress were normalised to range between zero and one, where one reflected the maximum value across all reef cells throughout each data set (observed and projected). Thus, we produced four single values of stress for each reef cell (Fig 1B). Disturbance regimes were delineated by identifying reef cells that fell within the upper and lower terciles of each stress measure calculated for the two time series. Upper and lower terciles (labeled as “high” and “low”) were chosen because reef cells attributed to those values were generally subjected to the most or least impacts on natural ecosystems [68]. To this end, each of the 428 reef cells (planning units) was allocated either to one of the 16 possible disturbance regimes or left uncategorized if the cell had any of the four values in the middle terciles. Of the 16 potential combinations, nine thermal-stress regimes were present in our study area and considered for management attention (Fig 4A and 4B).

#### Prioritization approach

To select priority areas for marine conservation, thereby enhancing our ability to promote resilience in a warming and uncertain future, we aimed to design a network of MPAs that addressed dynamic features of a seascape, such as the full range of thermal-stress regimes (see Fig 4A and 4B for regime definitions and S1 Fig for detailed characteristics). This approach targets areas that: (i) have relatively stable historical and/or future climates and are least affected by sporadic events (areas that are historical and/or future thermal refugia, and hence enhances species’ likelihood to persist) (regimes 1–4, 6); (ii) offer historical or future opportunities for increasing the capacity of species to respond to temperature rise through adaptive processes (regimes 1–4, 6, 8); (iii) do not experience stressful conditions in summer, both historically and in the future, which might be linked to extrinsic environmental factors such as oceanographic or topographic features, and prevent mortality (regime 5); (iv) are likely to have developed resistance given prior exposure to acute and/or chronic stress, coupled with relatively low corresponding future stress, which might maintain survival (regimes 2, 6–8); (v) require removal or mitigation of non-climate-related threats because of reduced ability to cope with additional chronic and/or acute stress in the future (regimes 3, 4, 9); (vi) are characterised by historical and/or future warming in non-summer periods, with reduced winter respites from summer-like conditions and make ecosystem more resistant or resilient to bleaching-stress events (regime 5); and (vii) are most likely to contain and are potentially dominated by disturbance-tolerant species and/or predicted to be thus characterised in the future, and can boost resilience to warming impacts (regimes 2–4, 6–9).

**Fig 5:**
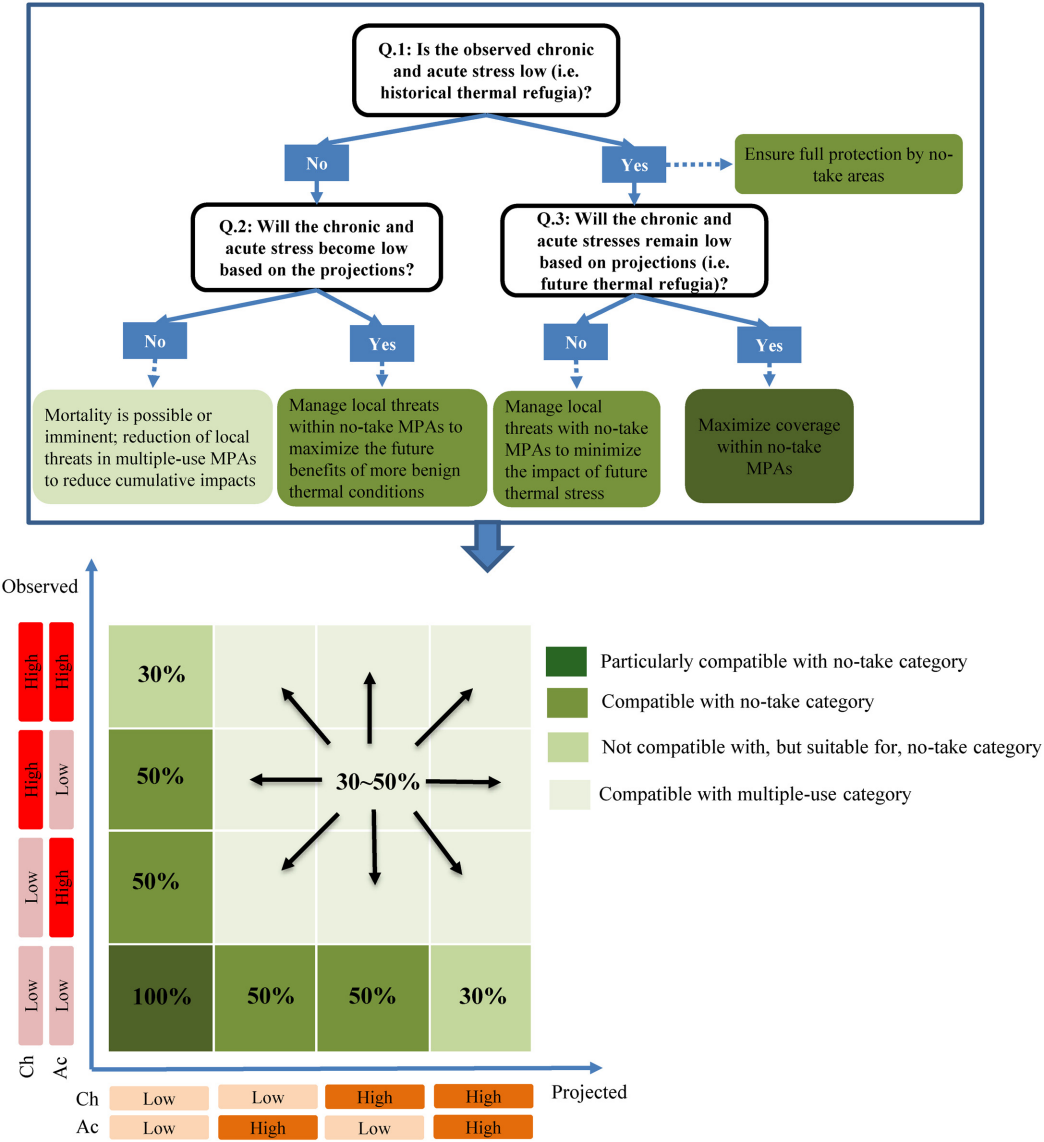
Decision tree for using information on chronic and acute stress derived from observed and projected data to formulate quantitative conservation objectives for warming disturbances. Ch = chronic stress, Ac = acute stress. Percentage values inside boxes in the bottom of the figure are prescribed (but indicative here) coverages by no-take and multiple-use MPAs.

Using the systematic conservation tool Marxan, we selected reef cells to achieve our conservation objectives for the nine regimes (Fig 4C). To formulate our conservation objectives, we reviewed recommendations from twelve papers ([19, 28, 45, 50, 53, 69–74]; see S1 Fig), offering guidelines for management of coral reefs under climate change or proposing methodological frameworks with conservation implications. We then used a decision tree to derive specific management requirements translated into quantitative objectives (Fig 5).

The suggested management type for achievement of objectives was defined by considering the historical or future ecosystem state in each regime. For example, areas that have experienced low levels of observed thermal stress or are projected to experience least future stress could retain naturally-functioning ecosystems, and should be more strictly protected (no-take MPAs). Conversely, reef cells that experienced high observed thermal stress or are projected to experience future stress might indicate some level of environmental degradation and are allocated within multiple-use MPAs. For thermal-stress regime 1 (see Fig 4C), the conservation objective was 100% of coverage within no-take areas, but objectives for other regimes are either 30% or 50%. The assignment of the MPA type to meet our conservation objectives was implemented as a *post-hoc* analysis and had no influence on the selection of priority areas in Marxan. Our conservation objectives are indicative and will need to be refined adaptively as knowledge accumulates on the management requirements of regimes.

Spatial prioritization in Marxan was repeated 100 times, and final conservation planning scenarios were obtained after 10 million iterations. We set a high penalty value to each thermal regime to ensure that all objectives were fully achieved. We ran two scenarios, one ignoring existing MPAs to identify their coincidence with areas selected at lowest possible cost (measured in this case by total area of reef cells) and one mandating protection for the existing MPAs, and therefore serving as a gap analysis (Fig 1C). For the second scenario, the reef cells coinciding spatially with existing MPAs (n = 60) were locked in for the analyses. For both scenarios, we recorded the best solution and selection frequencies of reef cells.

## Results

### Chronic thermal stress

**Fig 6:**
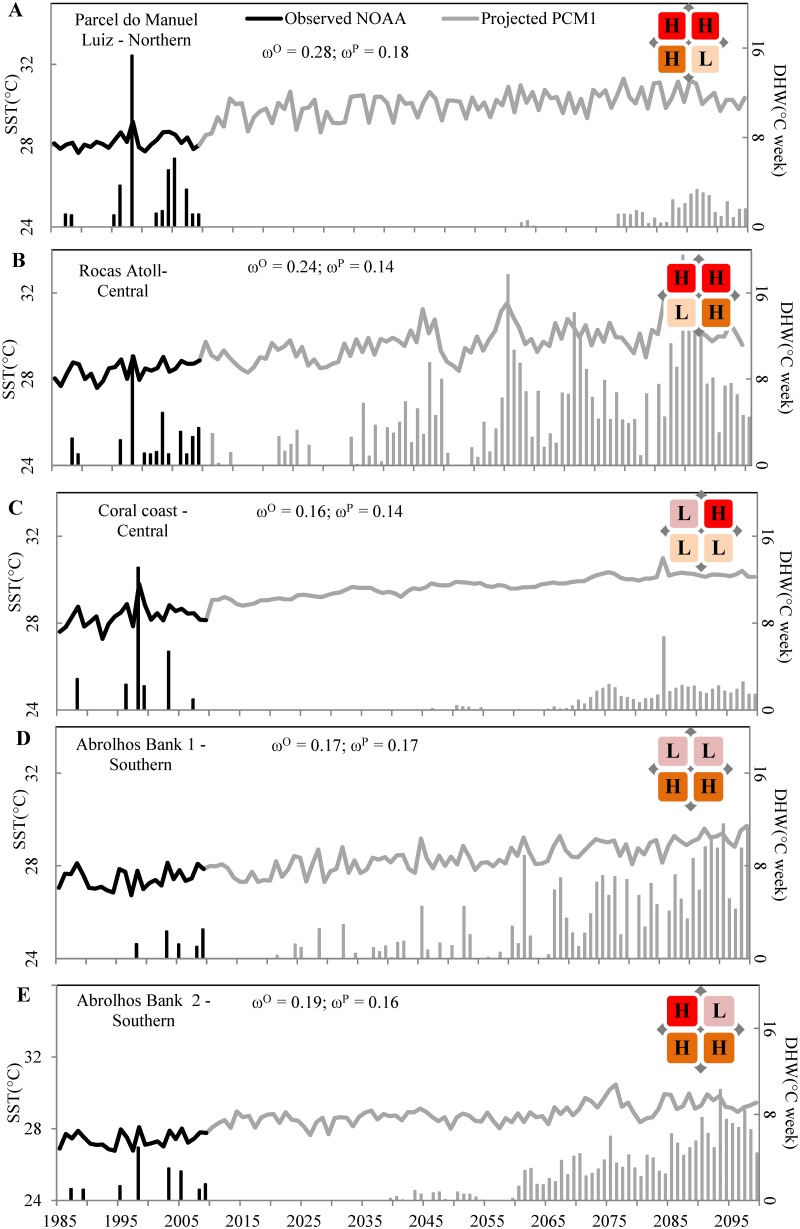
Annual maximum SST and Degree Heating Weeks (DHWs) for five reef cells within different thermal-stress regimes. Approximate locations of the five cells are shown as a-e in Fig 2A. Observed data (satellite NOAA) are shown by black solid lines (SST values) and filled bars (DHWs) while projections (GCM PCM1 output) are shown by gray solid lines (SST values) and filled bars (DHWs). Warming trends (in °C per decade) are shown for observed (ɷ^O^) and projected (ɷ^P^) time series. The thermal-stress regime allocated to each reef cell is indicated in the top right of each graph, and defined in Fig 3.

The observed and projected SST patterns identified an overall warming trend throughout our study area; there were no instances of observed nor projected of cooling trends (Fig 2B and 2C). To illustrate variations in SST characteristics, we selected five reef cells (shown in Fig 2A) within different thermal-stress regimes (Fig 6). Across the historical time series, warming rates ranged from 0.098°C to 0.280°C decade^–1^ (average: 0.19°C decade^–1^). Compared with observed satellite data, the projected rate of SST rise was slower through the 21st century PCM predictions (Fig 2C), and the range of projected trend values was smaller than observed ones within our study area (from 0.12 to 0.18°C decade^–1^; average: 0.15°C decade^–1^). However, it is notable that the time period through which trends were calculated differed nearly four-fold. While recent trends could accurately represent longer-term historical trends, they might also be influenced by ocean variabilities of multidecadal periodicity (e.g., the Atlantic Multidecadal Oscillation, 60–70 year period, [75]) that, depending on the phase through the calculation period, can enhance or diminish the short-term trend.

Although SST rose most rapidly in reefs closer to the equator (Figs 2B and 6A), reefs situated further south and in the central of the study area (Figs 2B and 6B–6E) also warmed quickly. Projected warming was more pronounced over the southern and northern reef cells than in the central sector. Even after suppressing SST variance associated with year-to-year variability induced by the ENSO cycle and seasonal variability, we detected greater variability in the most northern and southern reefs in our study area (Fig 6). Only 6.3% of the reef cells (all from the southern sector) had projected warming rates greater than observed trend.

### Acute thermal stress

**Fig 7:**
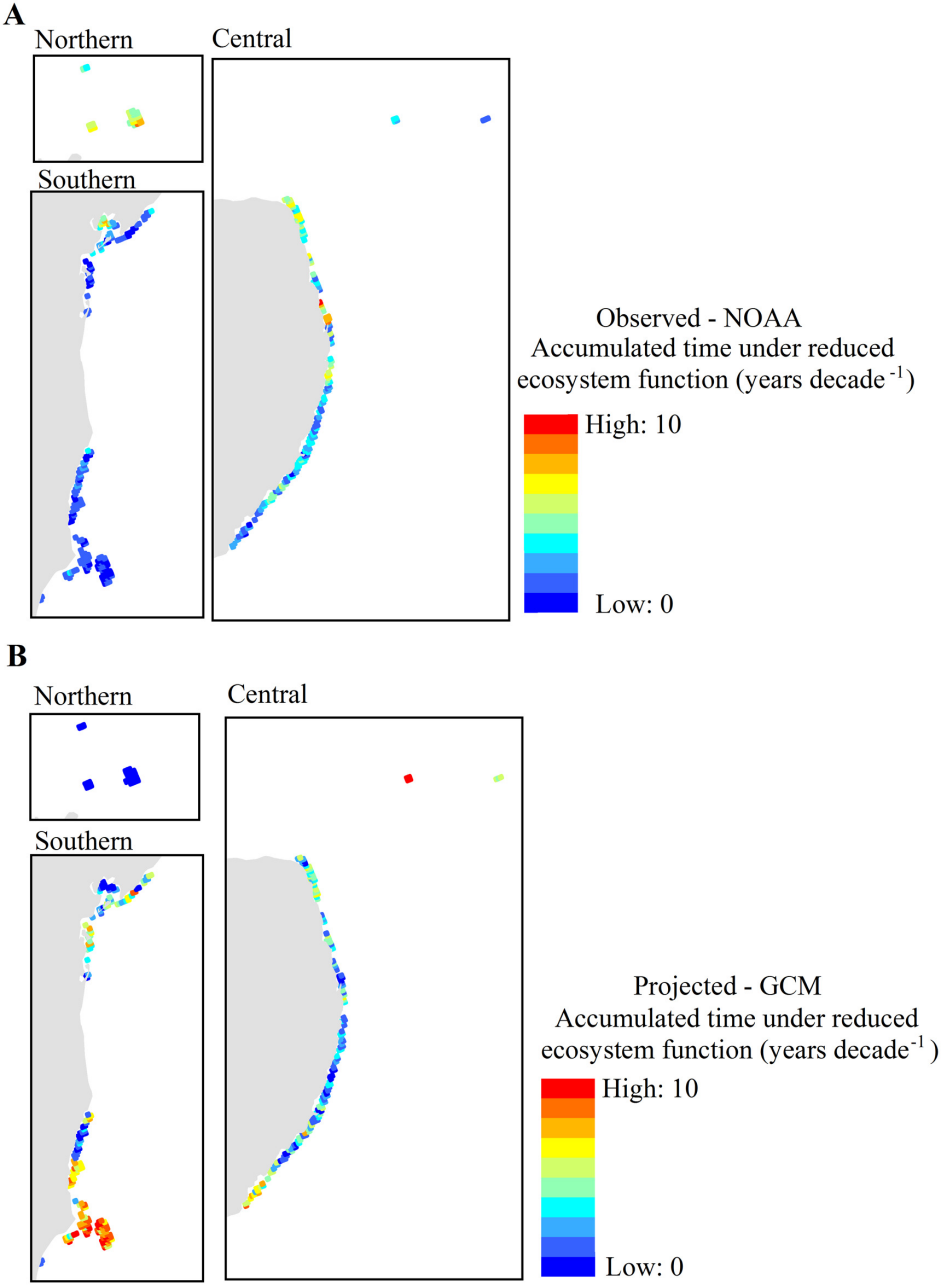
The acute thermal stress metric. Accumulated time for which the ecosystem is under reduced ecosystem function from acute stress events for all reef cells according to observed (A) and projected (B) time series. Times are derived from the logistic function used to relate intensity of acute stress events to recovery time (Fig 3) and summed through each time-series and presented as years per decade. Panels for reefs in the northern, central, and southern sectors of the study area correspond to insets in Fig 2A.

While projected warming rates in the future were lower than in recent historical data, an increase in the acute stress metric associated with bleaching-level stress events was evident for many reef cells located in the central and southern sectors (Figs 6 and 7). Nearly 45% of reef cells were projected to face a greater proportion of time under reduced ecosystem function following acute events than historically observed. Accumulated time under reduced ecosystem function across reef cells ranged from 0.6 to 10.0 years decade^–1^ for the observed time series (average: 1.93 years decade^–1^) and from 0.3 to 10.0 years for the projected GCM model (average: 2.59 years decade^–1^). Most reef cells had annual maximum DHW values exceeding 4°C-weeks in at least one year of both datasets (87% and 82% of reef cells in the observed and projected time series, respectively) over the whole planning time window (S2 Fig). The observed data indicated that only 3.9% of reef cells had been exposed to two or more bleaching-level events (DHW exceeding 4°C-weeks) per decade, but inferences from PCM model output indicate that 28% of reef cells were projected to exceed this event frequency (S2 Fig).

The acute stress metric through the observed data shows a spatial pattern of increased values towards the northern sector (Fig 7A). In contrast, reefs in the southern are projected to experience the greatest time under reduced ecosystem function in the future (Fig 7B). An exception to this general pattern is projected for the Rocas Atoll (offshore in the very north of the central sector, see also Fig 6B), where the most severe projected stress (19.4°C-weeks) will exceed that experienced historically (10.0°C-weeks). Southern reefs are projected to experience the greatest time under reduced ecosystem function because they are almost the only ones to have bleaching-level stress events exceeding the suggested threshold of two events per decade and thus leading to reef degradation (S2 Fig). In contrast, northern reefs are projected to maintain event frequencies similar to that recently observed through the remainder of the century (S2 Fig). Rocas Atoll is exceptional again because disturbances are predicted to occur at a rate approaching five per decade.

### Thermal-stress regimes and conservation objectives

**Fig 8:**
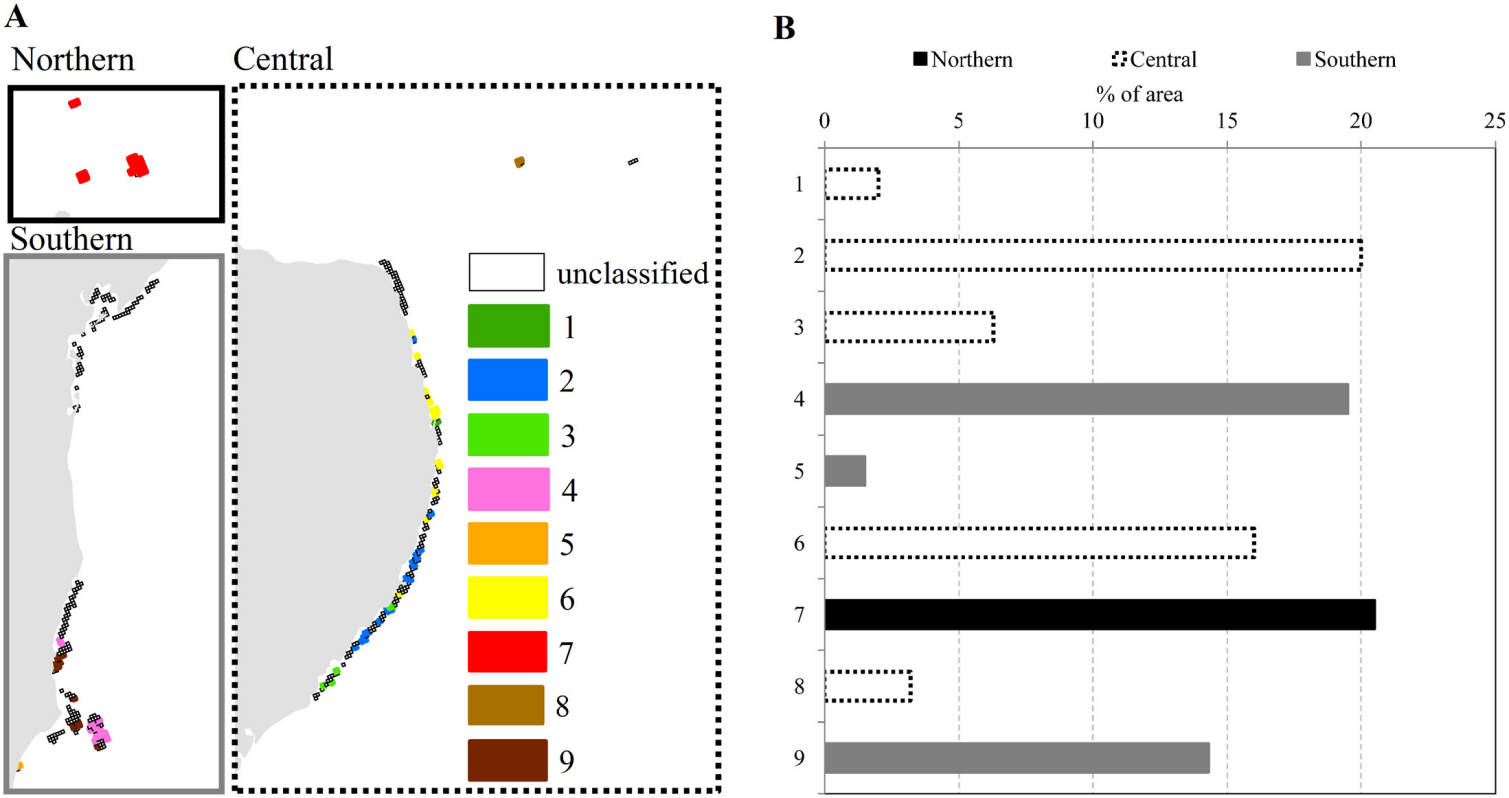
Distribution of thermal-stress regimes across study area. (A) Reef cells to which the nine regimes were allocated. Empty (black outlined) cells are unclassified because they have middle-tercile values for at least one of the four variables used to classify regimes. Labels for thermal regimes match those in Fig 4. Views for reefs in the northern, central, and southern sectors correspond to insets in Fig 2A. (B) Areal coverage of each thermal-stress regime as a percentage of the total of all reef cells allocated to regimes (n = 101). Each regime was encapsulated within a single sector.

Approximately 24% of reef cells (101 of 428) fell within one of our nine thermal-stress regimes, the remaining cells having middle-tercile values for at least one of the four variables used to classify regimes. Descriptive statistics for all metric values used to formulate regimes are shown in S1 Table. The cells allocated to regimes were distributed across all three sectors of our study area (Fig 8A). The most extensive regimes occurred in those areas subjected to recent bleaching-level stress events but with increased potential ability to survive future stress (high observed acute stress and low projected acute stress). These were regimes 2, 6, and 7, accounting for about 56% of the total reef cells assigned to regimes (Fig 8A and 8B). Examples of these regimes were mostly located on isolated reefs and near-shore banks off the coast in the central sector. Regimes that require management at local scales to avoid increased mortalities resulting from non-climate-related threats were also well represented. These regimes (3, 4 and 9) accounted for about 39% of all allocated reef cells (Fig 8A and 8B). These regimes were mostly represented in the southern sector and included the outer reef arc in the Abrolhos region. Only 2% of assigned reef cells were in regime 1, with minimal impacts from observed and projected chronic and acute stress (Fig 8B). These historical and future thermal refugia were inshore isolated bank reefs located off the central coast (Fig 8A). Areas projected to have higher rates of coral mortality from future acute stress, and therefore requiring local management interventions to mitigate impacts, include bank reefs forming the coastal arc of the Abrolhos Bank and the Rocas Atoll (regimes 4, 8 and 9 in Fig 8A).

The current system of MPAs achieved four of our nine conservation objectives (Fig 4C) considering both coverage and type of management. There were substantial shortfalls in achieving objectives for historical and/or future thermal refugia (regimes 1, 2, 3 and 6). Although regime 3 occurred widely in multiple-use areas (>80% of these areas), our objective for this regime was 50% coverage by no-take zones. A key finding of our gap analysis was that many regimes that could be managed inside multiple-use areas (less restrictive types of MPAs) achieved their objectives through coverage by no-take zones (Fig 4C).

**Fig 9:**
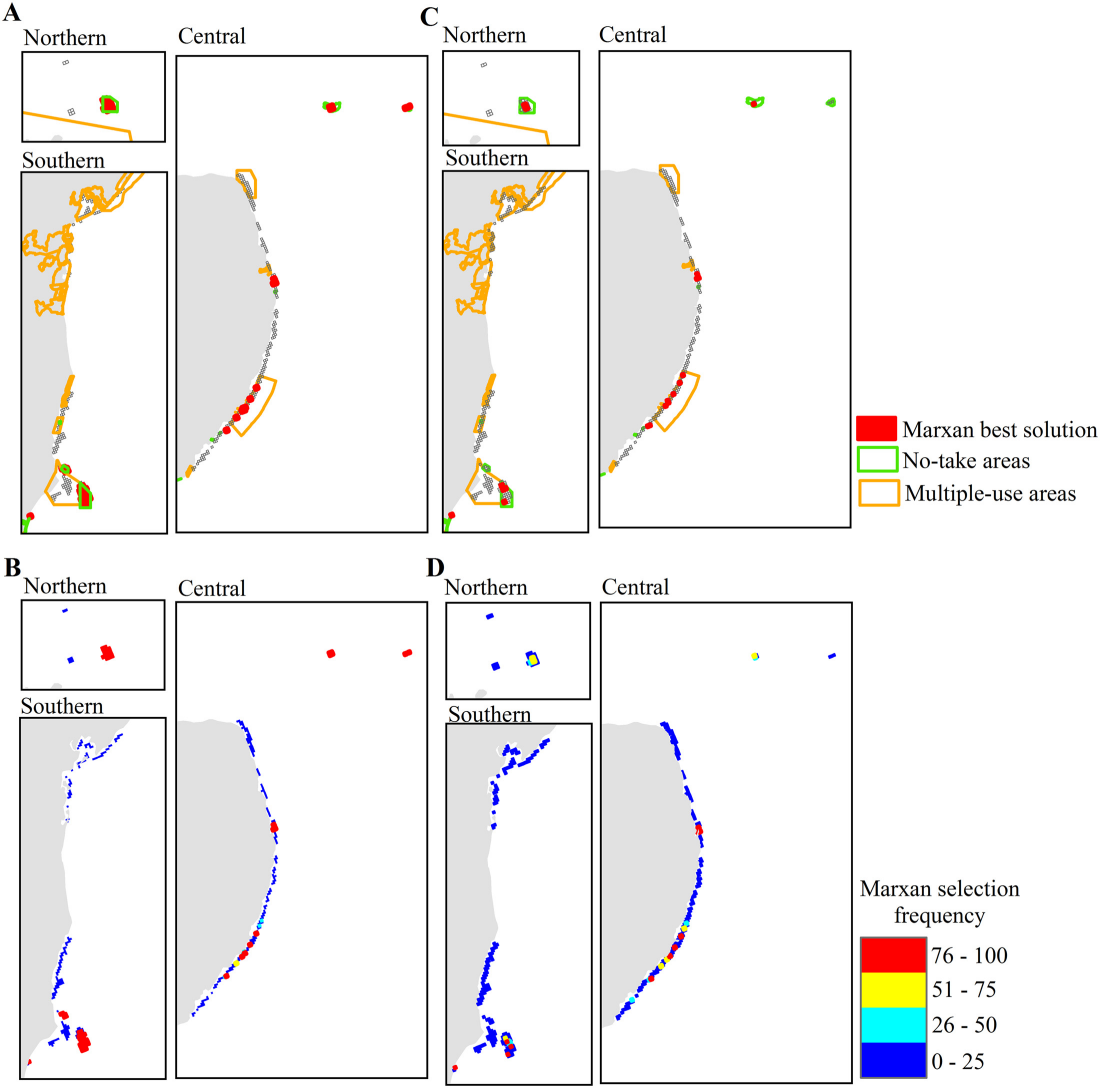
Spatial prioritization of coral reefs in Brazil based on our conservation objectives for incorporating warming impacts. Maps show best solution and selection frequency Marxan outputs when selection of reef cells coinciding with existing MPAs was mandatory (A and B, respectively) or optional (C and D, respectively). Views for reefs in the northern, central, and southern sectors of the study area correspond to insets in Fig 2A.

Requiring that existing MPAs be selected in the spatial prioritization analysis identified 16.8% of reef cells (72 of 428) to achieve the prescribed objectives (Fig 9A). All but twelve of these cells were in existing MPAs; however, some of these cells (n = 14) were within multiple-use MPAs and so did not contribute to the achievement of objectives requiring coverage by no-take areas. In contrast, without the requirement for existing MPAs to be part of the solution, the objectives were met with 9% of reef cells (Fig 9C). In this analysis, few reef cells appeared to be substantially more important than others (only 11 of the 428 reef cells had a selection frequency of 100%, Fig 9D). These 11 reef cells were mostly located in the central sector and included the two reef cells within regime 1.

## Discussion

Mitigation and avoidance of warming impacts are challenging issues for MPA planning. Designing effective systems of MPAs will require explicit management objectives and approaches that account for shifts in climate disturbances over time. Using chronic and acute thermal-stress metrics based on observed and projected SST data, we explored ways of assessing the adequacy of an MPA system under current and future climate circumstances when MPA boundaries were not spatially or temporally flexible. We showed that the waters of all coral reefs in Brazil have warmed and will continue warming. Events of acute temperature stress, and associated bleaching and potentially mortality, are expected to increase in frequency and intensity on the majority of the reefs studied. After setting explicit management objectives for reef cells in different temperature regimes, we demonstrated that the existing system of MPAs has important shortfalls, including unmet conservation objectives for historical and/or future thermal refugia. Some of the under-represented reef cells are spatially very restricted along Brazil’s central coast.

### Retrospective time-series

We found that Brazilian coral reefs were warming faster than the global average of 0.17 ± 0.05°C decade^–1^ [60], but less quickly than the averages for the Caribbean province of 0.29°C decade^–1^ [58] and the Coral Triangle of 0.2°C decade^–1^ [76]. Thus, the Western Atlantic coast might provide more favorable environmental conditions than other coral-reef regions for adaptation of the most thermally-sensitive species during global warming. Although climate change might be occurring more quickly than the rate at which most species can effectively respond through local adaptation or migration across seascapes [7, 14, 77], an urgently-needed extension of this and previous studies is a systematic comparison between coral-reef provinces, identifying areas globally where changes in climate are consistently attenuated. Secure protection of areas with low chronic stress might provide many opportunities for species persistence.

While recent instances of coral bleaching in our study area have been correlated with warming sea temperatures [37], there have also been mismatches between the timing of bleaching phenomena in some of our reef cells and major global events related to the periodic occurrence of ENSO variability (e.g., [13, 32, 37, 78]). Because bleaching events in Brazil (as elsewhere) are also driven by interactions with other stressors, such as eutrophication and sedimentation, a more comprehensive evaluation is needed for assessing exposure to other key factors that reinforce or reduce thermal stress (see Maina et al. [79]). Although considering different types of interactions between local and global stressors is important for management [80], assessing their combined impact is rarely straightforward. It can be technically demanding, requiring collection of datasets for environmental factors other than temperature, as well as better understanding of ecological consequences of interactions. While our work is focused on warming impacts, it provides management insights that are beneficial to advance reef conservation and decision-making by pinpointing areas where fine-resolution information on local stressors is particularly important.

The observed patterns of bleaching-level stress indicate that our study area has been exposed to DHW levels similar those of other areas across the globe [54, 81, 82]. Temporal mismatches between bleaching events observed in some of our reef cells and those in other reef provinces exposed to similar levels of acute thermal stress might also be explained by the lack of systematic effort in the reporting of bleaching in the western Atlantic Ocean. Bleaching data are essential for a better understanding of patterns of bleaching impact in response to thermal stress [83] and could be used to validate bleaching thresholds such as ours with information that is species- or site-specific. The available evidence indicates that not all corals respond identically to thermal stress; sensitivities vary substantially across species [14]. Background SST variability can also influence the thermal sensitivity of coral communities [82].

### Prospective time-series

PCM1 predictions of warming impacts in the period 2010–2099 demonstrate that, although the rate of warming is less than in recent decades, an incremental increase in the frequency and severity of bleaching-level events associated with massive mortality is expected for the majority of reefs in the southern sector. While such models provide an initial assessment of future vulnerability to thermal stress, PCM1 is among the models with lowest sensitivity; for example, for a doubling of CO_2_, the projected temperature increase is only 1.5°C [50]. Consequently, our results for bleaching-level events are likely to be underestimated. Importantly, the spatial distribution of metrics derived from projected time series did not coincide with those based on the historical dataset. Therefore, our findings indicate that MPA designations that ignore predictions for thermal stress might have little ability to adequately capture future distributions of thermal-stress regimes.

While we attempted to minimize the influence of other sources of variability in the future climate by selecting only one emission scenario, Makino et al. [84] have argued that priority should be given to areas that are selected consistently across all available scenarios as a way to identify no-regrets sites for conservation. From this point of view, one way forward is to establish robustness of results by examining the extent to which different scenarios of greenhouse-gas emissions would affect the spatial delineation of thermal regimes. By doing so, it might be possible to find a consensus among climate forecasts and shed light on the upper and lower bounds of uncertainty related to climate projections that will help to predict future conditions more accurately.

Although assembling a set of GCMs could offer a more comprehensive analysis of SST projections, we opted to use only one model output because different GCMs vary in their ability to correctly capture some aspects of SST, such as the amplitude of the seasonal cycle and variability due to El Nino-Southern Oscillation (ENSO) [51, 85, 86]. It is important to note that the assembly of multi-model predictions is very data-intensive and beyond the scope of this study, which aimed primarily to illustrate the potential benefits of incorporating modeled projections into conservation objectives.

Despite the employment of only one scenario and model output, our work demonstrates one ecologically meaningful way of interpreting and combining warming projections with reserve design. Although including projected changes in climate into MPA design carries inherent uncertainties [87], there is an urgent need to integrate proactive approaches within conservation plans to better understand future states and reduce the risk of poor conservation outcomes [50, 88, 89]. While we acknowledge assumptions in the ecological components of our modeling, model-based uncertainties can be reduced by the adoption of an adaptive planning framework for conservation [90]. The potential refinements to our current procedures would increase their ecological relevance and enhance the functional capability of areal classification by thermal-stress regime. The refinements can also be readily accommodated within our framework as more refined information becomes available.

### Conservation objectives for dealing with warming impacts

Conservation is unlikely to be successful or efficient in the long term if shifts in climate disturbances are not considered. Our results show that, although the current system of MPAs incorporates some of our warming-disturbance regimes, other important areas need protection through consideration of current and future thermal-stress regimes. While there is increasing pressure for evaluation of protected areas with respect to their intended objectives [91], systems of MPAs designed to represent static features might fail to meet objectives for spatially- and temporally-dynamic phenomena [28, 92]; it is therefore necessary to incorporate dynamic phenomena into the process of objective setting. Prioritization through the formulation of quantitative and well-defined objectives in combination with a spatially- and temporally-explicit methodology for planning with ecologically-informed parameters provides a best approach to planning for dynamic threats [18].

Calls to address climate-related disturbance by increasing MPA size or replicating features of interest in widely-spaced MPAs are widespread [18]. These guidelines reflect the difficulty of understanding of ecosystem responses to environmental change and to formulate quantitative objectives accordingly. Also, implementation of these general and usually qualitative recommendations might be impractical in real-world situations where there are severe spatial constraints on the extent of MPAs. Furthermore, MPA expansion is difficult to justify without well-argued objectives. We have improved upon more general recommended strategies for addressing climate change by proposing a methodological template to address specific warming-related variables to develop conservation objectives underpinned by ecological information. This study also demonstrated the value of combining retrospective information that might be valuable over the short term as a bet-hedging strategy (see Chollett et al. [24]) with projected SST to delineating thermal-stress regimes. As borne out by our analysis, current patterns of SST anomalies might not necessarily be indicative of the future [19, 28, 83].

In addition to using both historical and projected SST to better respond to warming impacts in marine planning, our conceptualization of objectives offers at least two other improvements on previous approaches. First, it acknowledges that many management types are needed for planning in the context of climate change [13, 93, 94]. With information on current and future exposure to warming disturbances, managers will be more able to identify areas requiring local actions for controlling non-climatic threats and will allow a broader temporal perspective when assessing the required level of active management and the most suitable MPA category under conditions of climate warming.

Second, our spatial prioritization was also framed such that severity of warming impacts triggered long-term conservation objectives. All previous studies have taken one of three approaches in the definition of timeframes: (i) calculating geometric means for predictions at arbitrary temporal intervals [8]; (ii) using midpoints or endpoints in the time series for fixed assessments as benchmarks [77, 84, 92, 95]; or (iii) including a large number of time steps in a data-intensive approach [26]. While we accounted for the whole time series for predicting shifts in disturbance regimes to estimate long term degradation rate, we also addressed the situation of temporally-static MPAs being preferable due to ease of implementation. Since there might be legislative, political, or implementation challenges in the creation of dynamic MPAs [92, 96, 97], our proactive MPA design provides an option for planners to address future states with static MPA boundaries. Although there are several limitations related to spatially fixed reserves (see Alagador et al. [98]), dynamic conservation planning involving selection of new MPAs and removal of others must be viewed with caution if this will risk undermining the integrity of the remaining MPA system by weakening protection to facilitate extractive uses.

SST data and projections provide only one layer of information when informing decisions about MPA placement. Including spatially-explicit data on socioeconomic variables [99] species occurrences [100], and dynamic factors relating to connectivity [101] will broaden our methods and determine whether the spatial patterns of conservation that emerge from our methods would be changed by other considerations. Importantly, we did not account for other threats to determine where multiple stressors occur concurrently, which could lead to incorrect identification of sites requiring management. The combined effects of multiple stressors need to be further assessed, particularly because the impacts of climate warming are compounded by those arising from local human activities, over which managers have direct influence [50].

It might also be possible to incorporate other significant impacts of climate change on coral reefs, such as ocean acidification and sea-level rise (see McLeod et al. [83]), into MPA design to fully understand how future ocean conditions can be accommodated by conservation planning. Incorporating only metrics of thermal stress means that our framework should be regarded as a first step for conservation planners to deal with climate-change impacts.

Our classification of regimes offers insights into coral-reef conservation, although we believe that this approach can be adapted to inform conservation of other marine ecosystems. For example, studies in rocky reefs, kelps, seagrasses, and mangroves have also identified climate-warming effects on ecosystem functioning [102–105]. Such effects could be interpreted for conservation planning into a similar framework to ours, as could regimes integrating information across other impacts related to climate change.

## Conclusions

Our aim was to help decision-makers in prioritizing areas considering long-term vulnerabilities to climate warming. We developed an approach to MPA design with a spatially- and temporally- quantitative procedure that accounts for historical and projected sea surface temperature. We projected, on the basis of GCM modeling, that bleaching-level stress in Brazil will tend to increase while the rate of warming appears to decrease, and interpreted these changes relative to recovery times of coral communities. We also determined the extent to which existing Brazilian MPAs achieve our conservation objectives in the face of dynamic threats and showed how additional MPAs might be designed to account for both historical and future thermal stress. Using a prospective approach such as ours is advantageous when anticipating shifts in predicted disturbance regimes in cases where temporally-static MPAs are more feasible than dynamic ones that can be shifted to accommodate future conditions.

## Supporting Information

S1 Fig Characteristics of thermal-stress regimes. Regimes are made up of different combinations of metrics for chronic (Ch) and acute (Ac) stress derived from observed (Ob) and projected (Pr) time-series. Based on supporting literature, rationales for their conservation are presented.
(DOCX)

S2 Fig Intensity and frequency of bleaching-level stress (acute) events. The highest annual maximum DHW based on observed (A) and projected (B) SST values as a indicator of intensity of acute events. Average number of bleaching-level stress events (when DHW > 4°C-weeks) per decade as an indicator of the frequency of acute events, derived from observed (C) and projected (D) SST time-series. Views for reefs in the northern, central, and southern sectors of the study area correspond to insets in Fig 2A.
(DOCX)

S1 File Additional methods. Description of methods involved in the bias correction and downscaling. To illustrate our methods, Figure A depicts annual maximum SST for five reef cells across study area. Observed data (satellite NOAA) are shown by black solid lines (1985–2009) while projections (retrospective and future) are shown by black dotted lines for raw GCM PCM1 output and gray solid lines after bias removal (corrected values). Approximate locations of the five cells (a-e) are shown in Fig 2A. Spearman correlation (r) between annual maximum SST from satellite data and PCM1 after bias removal for the retrospective “training period” (1985–1999, indicated by the shading area in the graph) are presented for five reef cells.
(DOCX)

S1 Table Descriptive statistics for all normalised metrics used to formulate thermal stress regimes. (DOCX)
