# Supplementary material for: Conservation Planning for Coral Reefs Accounting for Climate Warming Disturbances
Source: PLoS One. 2015 Nov 4;10(11):e0140828. doi: 10.1371/journal.pone.0140828 (PMC4633137; doi:10.1371/journal.pone.0140828)

**S1 Fig. Characteristics of thermal-stress regimes.** Regimes are made up of different combinations of metrics for chronic (Ch) and acute (Ac) stress derived from observed (Ob) and projected (Pr) time-series. Based on supporting literature, rationales for their conservation are presented.


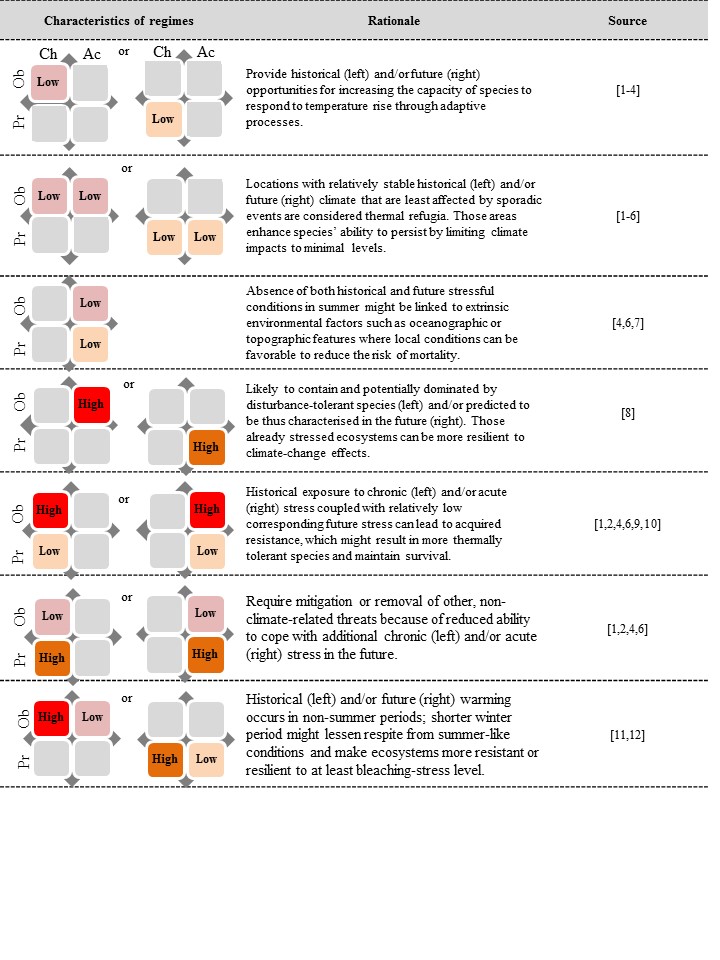


**Reference**

1. Hansen L, Hoffman J, Drews C, Mielbrecht E (2010) Designing climate-smart conservation: guidance and case studies. Conservation Biology 24: 63-69.
2. Keller BD, Gleason DF, McLeod E, Woodley CM, Airamé S, Causey BD, Friedlander AM, Grober-Dunsmore R, Johnson JE, Miller SL (2009) Climate change, coral reef ecosystems, and management options for marine protected areas. Environmental management 44: 1069-1088.
3. Selig ER, Casey KS, Bruno JF (2010) New insights into global patterns of ocean temperature anomalies: implications for coral reef health and management. Global Ecology and Biogeography 19: 397-411.
4. West JM, Salm RV (2003) Resistance and resilience to coral bleaching: implications for coral reef conservation and management. Conservation Biology 17: 956-967.
5. McCook LJ, Almany GR, Berumen ML, Day JC, Green AL, Jones GP, Leis JM, Planes S, Russ GR, Sale PF (2009) Management under uncertainty: guide-lines for incorporating connectivity into the protection of coral reefs. Coral Reefs 28: 353-366.
6. McLeod E, Salm R, Green A, Almany J (2009) Designing marine protected area networks to address the impacts of climate change. Frontiers in Ecology and the Environment 7: 362-370.
7. Ban NC, Pressey RL, Weeks S (2012) Conservation Objectives and Sea-Surface Temperature Anomalies in the Great Barrier Reef. Conservation Biology 26: 709-809.
8. Côté IM, Darling ES (2010) Rethinking ecosystem resilience in the face of climate change. PLoS Biology 8: 1-5.
9. Selig ER, Casey KS, Bruno JF (2012) Temperature‐driven coral decline: the role of marine protected areas. Global Change Biology 18: 1561-1570.
10. van Hooidonk R, Maynard JA, Liu Y, Lee S‐K (2015) Downscaled projections of Caribbean coral bleaching that can inform conservation planning. Global Change Biology, doi: 10.1111/gcb.12901
11. Heron SF, Willis BL, Skirving WJ, Eakin CM, Page CA, Miller IR (2010) Summer hot snaps and winter conditions: modelling white syndrome outbreaks on Great Barrier Reef corals. PloS one 5, e12210.
12. Maynard J, van Hooidonk R, Eakin CM, Puotinen M, Heron SF, Garren M, Lamb J, Williams G, Weil E, Willis B, Harvell CD (2015) Climate projections of conditions that increase coral disease susceptibility and pathogen virulence. Nature Climate Change (in press).


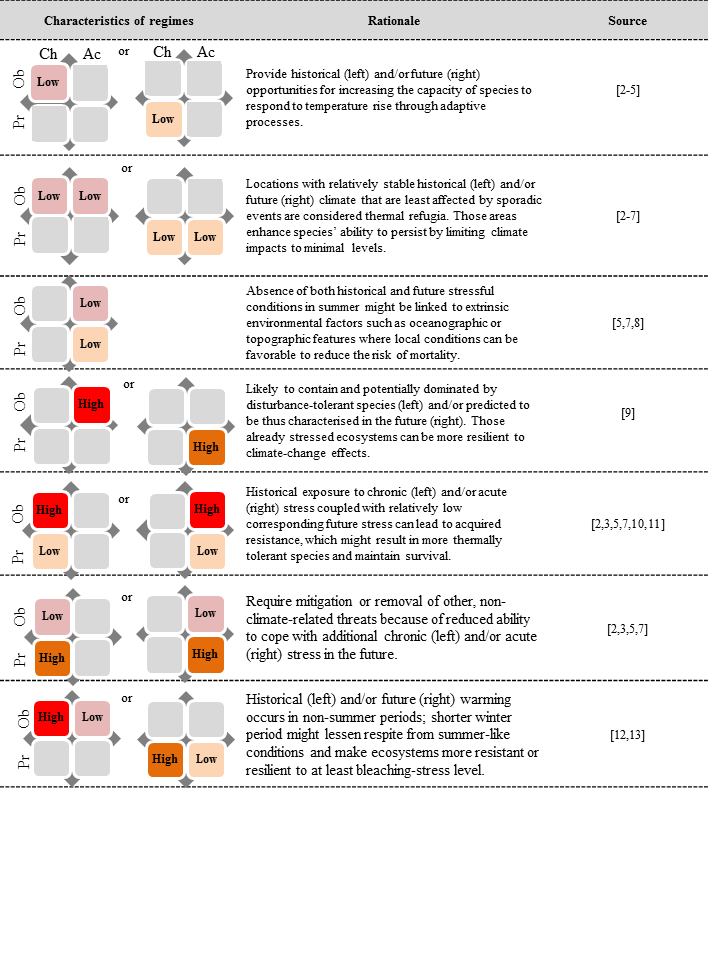

Supplement: S1 Fig — Regimes are made up of different combinations of metrics for chronic (Ch) and acute (Ac) stress derived from observed (Ob) and projected (Pr) time-series. Based on supporting literature, rationales for their conservation are presented. (DOCX) [file pone.0140828.s001.docx]
