# Supplementary material for: Conservation Planning for Coral Reefs Accounting for Climate Warming Disturbances
Source: PLoS One. 2015 Nov 4;10(11):e0140828. doi: 10.1371/journal.pone.0140828 (PMC4633137; doi:10.1371/journal.pone.0140828)

**S2 Fig. Intensity and frequency of bleaching-level stress (acute) events.** The highest annual maximum DHW based on observed (A) and projected (B) SST values as a indicator of intensity of acute events. Average number of bleaching-level stress events (when DHW > 4) per decade as an indicator of the frequency of acute events, derived from observed (C) and projected (D) SST time-series. Views for reefs in the north, centre, and south of the study area correspond to insets in Fig. 2A.


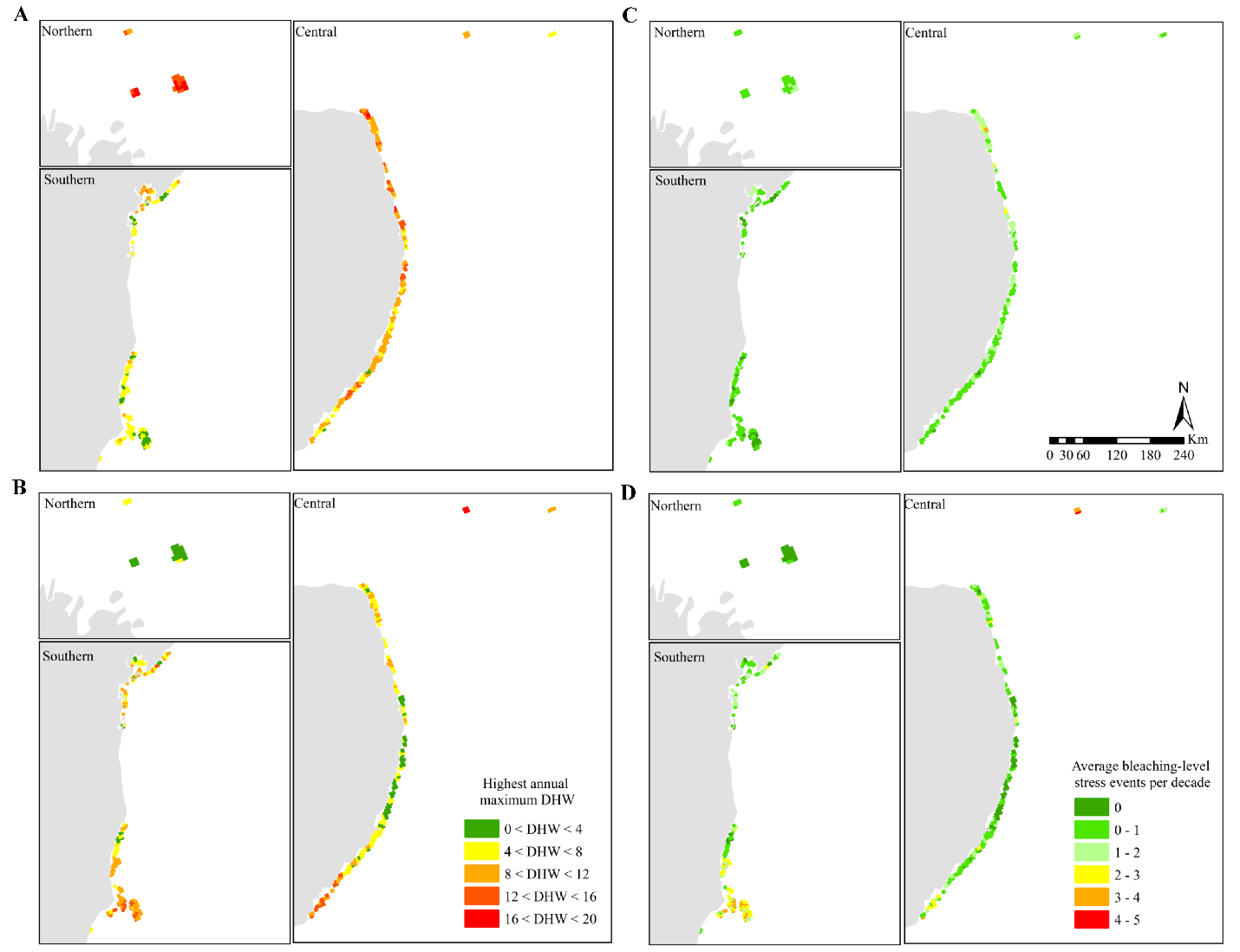

Supplement: S2 Fig — The highest annual maximum DHW based on observed (A) and projected (B) SST values as a indicator of intensity of acute events. Average number of bleaching-level stress events (when DHW > 4°C-weeks) per decade as an indicator of the frequency of acute events, derived from observed (C) and projected (D) SST time-series. Views for reefs in the northern, central, and southern sectors of the study area correspond to insets in Fig 2A. (DOCX) [file pone.0140828.s002.docx]
