# Supplementary material for: Conservation Planning for Coral Reefs Accounting for Climate Warming Disturbances
Source: PLoS One. 2015 Nov 4;10(11):e0140828. doi: 10.1371/journal.pone.0140828 (PMC4633137; doi:10.1371/journal.pone.0140828)
Supplement: S1 File — Description of methods involved in the bias correction and downscaling. To illustrate our methods, Figure A depicts annual maximum SST for five reef cells across study area. Observed data (satellite NOAA) are shown by black solid lines (1985–2009) while projections (retrospective and future) are shown by black dotted lines for raw GCM PCM1 output and gray solid lines after bias removal (corrected values). Approximate locations of the five cells (a-e) are shown in Fig 2A. Spearman correlation (r) between annual maximum SST from satellite data and PCM1 after bias removal for the retrospective “training period” (1985–1999, indicated by the shading area in the graph) are presented for five reef cells. (DOCX) [file pone.0140828.s003.docx]

### S1 File. Additional methods. Description of methods involved in the bias correction and downscaling.

Global projection of SST are produced at a coarse resolution and might over-represent SST means. We followed the method described by Dunne et al. [1] for bias correction through downscaling that will enable planners to undertake a conservation planning that is more informed. As standards for comparisons between actual and modeled SST in the validation procedures, we used monthly records for SST observational data obtained from NOAA AVHRR and hindcasts for GCM PCM1 outputs for a previous “training period” (1985–1999) of 20^th^ century simulations (20C3M). Initially, we generated a monthly climatology for both NOAA and PCM1 and calculated the difference between the maximum of each climatology as an anomaly to develop the mean-corrected SST, or${SST}^{MC}$. Rather than using binned SST data on a decadal scale, as originally suggested by Dunne et al. [1], we calculated the climatological data for 5-year periods of time because our training period spanned only 15 years. To normalize PCM1 variability to observations over the entire retrospective (the training period) and projected time frames, we calculated annual maximum SST (${SST}^{AM}$) and then the moving 5-year mean maximum SST (as 5-year-box-car smoothed values of), inserting median values into the start and end years of these periods. The variance-corrected SST (${SST}^{VC})$was calculated with the following expression:

${SST}^{VC}={SST}^{MC}-\left( {SST}^{AM}-{SST}^{TM} \right)\times\left( 1-{SST}^{NOAA\_GCM}/{SST}^{GCM} \right)$_1_

where, ${SST}^{MC}$is a mean-corrected SST (difference between the maximum for each climatology), ${SST}^{AM}$is the annual maximum, ${SST}^{TM}$ is the moving 5-year mean maximum, ${SST}^{NOAA\_GCM}$ and ${SST}^{GCM}$are the single 5-year maximum anomaly estimates for both NOAA and GCM datasets, respectively. These latter anomalies are the differences between the 5-year maximum and the climatological maximum for the 5-year periods. Anomalies were averaged to produce a single value for each dataset. Averages were applied only to those reef cells where ${SST}^{NOAA\_GCM}/{SST}^{GCM}$ was less than one to avoid adding variability where NOAA gave more variability than GCM. For model validation, we compared the climatology for the “training period” (1985-1999) provided by PCM1 and actual satellite data. We determined the correlation between predicted and observed SST values in a pairwise comparison. Values for Spearman correlation ranged from 0.7321 and 0.9875 (mean = 0.9237, median = 0.9058) with p-value < 0.005 (see Figure A for the correlation values for five reef cells).

**Reference**

1. Dunne JP, Stouffer RJ, John JG (2013) Reductions in labour capacity from heat stress under climate warming. Nature Climate Change 3: 563-566.

**

Fig. A. Annual maximum SST for five reef cells across study area**. Observed data (satellite NOAA) are shown by solid lines in black (1985-2009) while projections are shown by dotted lines for raw GCM PCM1 output and solid lines in gray after bias removal (corrected values). Approximate locations of the five cells (a-e) are shown as in Fig. 2A. Spearman correlation (r) between annual maximum SST from satellite data and PCM1 after bias removal for the “training period” (1985–1999, indicated by the shading area in the graph) are presented for five reef cells.
