# Supplementary material for: Conservation Planning for Coral Reefs Accounting for Climate Warming Disturbances
Source: PLoS One. 2015 Nov 4;10(11):e0140828. doi: 10.1371/journal.pone.0140828 (PMC4633137; doi:10.1371/journal.pone.0140828)
Supplement: S1 Table — (DOCX) [file pone.0140828.s004.docx]

**Table S1. Descriptive statistics for all metrics used to formulate thermal stress regimes**

|  | Chronic/Observed | Acute/Observed | Chronic/Projected | Acute/Projected |
| --- | --- | --- | --- | --- |
| First tercile | **0.414** | **0.080** | **0.276** | **0.056** |
| Third tercile | **0.544** | **0.267** | **0.580** | **0.171** |
| Mean | **0.511** | **0.212** | **0.475** | **0.163** |
| Median | **0.482** | **0.132** | **0.501** | **0.098** |
